# Social alienation among older adults with cognitive frailty: a latent profile analysis

**DOI:** 10.3389/fpubh.2026.1830537

**Published:** 2026-09-01

**Authors:** Songqing Zhao, Weiping Lu, Peng Lu, Li Zhou, Yan He, Yaohuan Sun, Zeen Wang

**Affiliations:** 1 Department of Geriatrics, The Affiliated Huaian No.1 People's Hospital of Nanjing Medical University, Huai'an, China; 2 Department of Endocrinology and Metabolism, The Affiliated Huaian No.1 People's Hospital of Nanjing Medical University, Huai'an, China; 3 School of Nursing, Fudan University, Shanghai, China

**Keywords:** cognitive frailty, heterogeneity, latent profile analysis, nursing assessment, older adults, social alienation

## Abstract

**Background:**

The rising prevalence of cognitive frailty among older adults necessitates a deeper understanding of its psychosocial consequences. Social alienation, a critical yet understudied dimension, may exhibit significant heterogeneity in this vulnerable population, with distinct subgroups potentially requiring tailored interventions. Elucidating these patterns could inform more targeted geriatric care strategies.

**Objectives:**

This study aimed to identify latent profiles of social alienation among older adults with cognitive frailty and examine the associations between sociodemographic and clinical factors and profile membership.

**Methods:**

A multicenter cross-sectional study was conducted from June to September 2025. A convenience sample of 968 older adults with cognitive frailty was recruited from four tertiary hospitals in Jiangsu Province, China. Latent profile analysis and multinomial logistic regression were performed on data collected using the General Alienation Scale, Perceived Social Support Scale, Geriatric Depression Scale, and Barthel Index.

**Results:**

Four distinct profiles of social alienation were identified: “Social Adaptation” (15.5%), “Mild Alienation” (23.5%), “Moderate Alienation” (26.5%), and “Severe Alienation” (34.5%). Depression was a significant risk factor associated with higher odds of belonging to all alienation profiles, whereas greater daily living abilities and perceived social support were protective. Notably, living with adult children (compared to living with a spouse) was an independent risk factor specifically associated with the “Severe Alienation” profile.

**Conclusions:**

Social alienation among older adults with cognitive frailty is highly heterogeneous, with a substantial proportion experiencing severe alienation. These findings suggest that routine screening for alienation could be a valuable addition to geriatric nursing assessments. Furthermore, our results provide an empirical foundation for future studies to develop and test targeted interventions, such as physical and psychological support programs for high-risk individuals, and to explore the feasibility of mobilizing peer support from well-adapted groups.

## 1 Introduction

With the acceleration of global population aging, cognitive frailty (CF) has emerged as a critical geriatric syndrome (1). Defined by the International Academy on Nutrition and Aging (IANA) and the International Association of Gerontology and Geriatrics (IAGG), CF refers to the coexistence of physical frailty and mild cognitive impairment in the absence of dementia (2). This syndrome is prevalent among community-dwelling older adults; recent systematic reviews estimate its pooled global prevalence at 10.3% (3), with the prevalence in the Chinese population reaching approximately 12.5% (4). A growing body of research confirms that CF not only severely threatens the health of older adults but also serves as an independent predictor of adverse outcomes, including falls, disability, reduced quality of life, increased hospitalization, dementia progression, and mortality (5, 6). For instance, older adults with CF face a 3.12-fold increased risk of all-cause mortality compared to their robust peers (7), and a 4-year prospective cohort study in Japan found that those diagnosed with CF at baseline had an 8.92-fold higher risk of developing dementia (8).

However, unlike the often irreversible trajectory of dementia, CF is widely regarded as a potentially reversible preclinical stage. A 6-year prospective cohort study by Tang et al. tracking the dynamic changes of CF status confirmed that a considerable proportion of patients can recover to a healthy state (9). Existing evidence suggests that multi-domain interventions—combining physical exercise, cognitive training, and nutritional support—can effectively improve both frailty status and cognitive function (10), presenting a vital “window of opportunity” for nursing professionals to prevent disability and cognitive decline.

Despite the well-documented physiological hazards of CF, its negative impact on psychosocial adaptation in older adults remains largely overlooked. One critical aspect is social alienation, defined as a subjective experience of feeling isolated, rejected, or disconnected from social networks. This experience may be particularly prevalent among older adults with CF due to multidimensional mechanisms (11). Physically, functional limitations such as reduced muscle strength and slow gait directly restrict opportunities for social participation (12, 13). Cognitively, deficits in memory and executive function can induce stigma and social anxiety, leading older adults to adopt defensive social withdrawal strategies to avoid interpersonal difficulties (14). Crucially, this alienation can create a vicious cycle: neuroimaging studies indicate that social isolation is independently associated with atrophy in key brain regions linked to memory (15), and loneliness significantly elevates the risk of incident dementia (16), thereby potentially accelerating the deterioration of CF itself. Therefore, understanding and breaking this reciprocal reinforcement between alienation and cognitive decline is an indispensable component of holistic nursing interventions for this population.

Previous research on social alienation in older adults with CF has predominantly adopted a “variable-centered” approach, such as regression analysis, which examines average associations between variables and implicitly assumes population homogeneity. However, older adults with CF exhibit significant heterogeneity in social resources, psychological resilience, and family support. Relying solely on aggregate-level analysis may obscure the unique characteristics of high-risk subgroups, leading to untargeted interventions. In contrast, Latent Profile Analysis (LPA), a “person-centered” approach, can identify distinct latent classes based on individual response patterns across multiple indicators, thereby overcoming these limitations. To our knowledge, no study has yet utilized LPA to explore the classification profiles of social alienation in this specific population (17).

Therefore, this study employed Latent Profile Analysis to uncover latent profiles of social alienation among older adults with cognitive frailty. Specifically, the aim of this study was to identify these latent profiles and examine the associations between sociodemographic and clinical factors and profile membership. By doing so, we seek to deepen the understanding of this complex phenomenon and provide empirical evidence for identifying high-risk subgroups and designing precise, targeted nursing interventions.

## 2 Methods

### 2.1 Participants and settings

A cross-sectional descriptive design was employed. The study followed the STROBE checklist as a reporting guideline (18). Data were collected from June to September 2025 across four tertiary general hospitals in Jiangsu Province, involving 968 older adults with cognitive frailty. Inclusion criteria were: (1) age ≥60 years; (2) meeting the diagnostic criteria for cognitive frailty, which was operationalized as the coexistence of physical frailty (assessed using the Fried Frailty Phenotype, with a score of ≥3 indicating frailty) and mild cognitive impairment [assessed using the Clinical Dementia Rating (CDR), with a score of 0.5 indicating mild cognitive impairment] in the absence of clinical dementia (19); and (3) conscious with basic communication abilities. To ensure data collection consistency across the four participating tertiary general hospitals, all investigators (uniformly trained clinical nurses) strictly followed a standardized operating procedure (SOP) for participant screening, interview protocols, and clinical assessments. Exclusion criteria included: (1) diagnosed dementia or severe mental illness; (2) severe visual or hearing impairments preventing cooperation; and (3) severe and unstable somatic diseases.

The sample size was estimated based on Kendall's rule of thumb, which suggests that the sample size should be 10 to 20 times the number of major variables. In this study, the Latent Profile Analysis (LPA) included 15 items from the GAS scale as explicit indicators, theoretically requiring a sample size of at least 300 cases. Additionally, following the recommendations of Nylund-Gibson et al., a sample size of at least 300 is required for LPA to ensure the convergence of model parameter estimation and the stability of results (20). Considering a potential 20% rate of invalid questionnaires or missing data, the sample size was appropriately expanded. Ultimately, 968 valid samples were included in the analysis, fully meeting the requirements for large-sample statistical analysis.

### 2.2 Data collection

The study was conducted after obtaining approval and assistance from the nursing departments of the respective hospitals. Data collection was performed by uniformly trained clinical nurses using face-to-face, one-on-one interviews. Prior to the survey, investigators detailed the study purpose, procedures, anonymity, and confidentiality principles to older patients meeting the inclusion criteria, and conducted the questionnaire survey after obtaining written informed consent. Considering that some older patients might have visual impairment or writing difficulties, investigators read the questionnaire items aloud in a neutral, objective manner for participants unable to fill it out independently, and checked the options based on their verbal responses, strictly avoiding leading language. Questionnaires were retrieved on the spot upon completion; investigators immediately checked for data completeness and requested participants to complete any missing items to ensure the validity rate of returned questionnaires.

### 2.3 Instruments

#### 2.3.1 General demographic questionnaire

This questionnaire was designed by the researchers and included the following variables: age, gender, education level, marital status, living arrangements, monthly income, medical payment method, comorbidity of chronic diseases, and history of falls.

#### 2.3.2 General alienation scale (GAS)

Social alienation was measured using the Chinese version of the GAS, originally developed by Jessor et al. (21) and adapted by Wu et al. (22). The scale consists of 15 items across four dimensions: self-alienation (3 items), alienation from others (5 items), suspicion (4 items), and meaninglessness (3 items). Each item is rated on a 4-point scale ranging from 1 (“strongly disagree”) to 4 (“strongly agree”). Total scores range from 15 to 60, with higher scores indicating higher levels of social alienation. The Cronbach's α coefficient for this scale in the present study was 0.770.

#### 2.3.3 Perceived social support scale (PSSS)

The PSSS, developed by Zimet et al. (23) and adapted into Chinese by Wang et al. (24), was used to assess the level of social support perceived by individuals. The scale comprises 12 items divided into three dimensions: family support (4 items), friend support (4 items), and support from others (4 items). Items are rated on a 7-point Likert scale ranging from 1 (“very strongly disagree”) to 7 (“very strongly agree”), with total scores ranging from 12 to 84. Higher scores indicate higher levels of perceived social support. The Cronbach's α coefficient for this scale in the present study was 0.835.

#### 2.3.4 Geriatric depression scale (GDS)

Depressive symptoms were assessed using the short form of the GDS developed by Sheik and Yesavage (25), which consists of 15 items. Participants respond “yes” (score 1) or “no” (score 0) to each item, with total scores ranging from 0 to 15. Higher scores indicate more severe depressive symptoms. The scoring criteria are as follows: 0–4 indicates normal, 5–9 indicates mild depression, and ≥10 indicates moderate to severe depression. Tang (26) verified the reliability and validity of this scale in the Chinese older adults. The Cronbach's α coefficient for this scale in the present study was 0.793.

#### 2.3.5 Barthel index (BI)

Activities of Daily Living were assessed using the BI developed by Mahoney and Barthel (27). The index includes 10 items such as grooming, bathing, dressing, feeding, and toileting. Total scores range from 0 to 100. The classification standards are: ≤ 40 indicates severe dependency, 41–60 indicates moderate dependency, 61–99 indicates mild dependency, and 100 indicates independence. Higher scores reflect better self-care ability.

### 2.4 Data analysis

Data analysis was performed using SPSS 26.0 and Mplus 8.3. Continuous variables with a normal distribution were expressed as mean ± standard deviation ($\bar{x}$ ± *s*), and group comparisons were conducted using one-way analysis of variance (ANOVA). Categorical variables were expressed as frequencies and percentages (*n*, %), and group comparisons were performed using the χ2 test. Latent Profile Analysis (LPA) was conducted using Mplus 8.3 to identify distinct subgroups of social alienation, with the 15 item scores of the GAS scale used as continuous indicators. Robust maximum likelihood estimation (MLR) was employed to estimate model parameters to account for any potential non-normality in the data. Missing data were handled using Full Information Maximum Likelihood (FIML) estimation, which allowed all available information from the 968 participants to be utilized without case deletion or introducing bias. In accordance with standard LPA conventions, local (item-level) conditional independence was assumed, meaning that the observed indicators were assumed to be independent of one another conditional on latent class membership. The optimal number of latent classes was determined based on a comprehensive evaluation of statistical fit indices, including the Akaike Information Criterion (AIC), Bayesian Information Criterion (BIC), sample-size adjusted BIC (aBIC), Entropy (>0.8), Lo-Mendell-Rubin likelihood ratio test (LMR-LRT), and Bootstrapped Likelihood Ratio Test (BLRT) (28).

Based on the results of the univariate analysis, variables with statistically significant differences (*P* < 0.05)—including marital status, living arrangements, social support, depression, and activities of daily living—were included as independent variables in a multinomial logistic regression model. Additionally, to rigorously control for potential confounding effects, theoretically relevant socio-demographic characteristics (age, gender, and education level) were forced into the multivariate model as covariates, regardless of their univariate significance. Prior to running the regression, multicollinearity among all independent variables was formally evaluated using the Variance Inflation Factor (VIF). All calculated VIF values were well below the conservative threshold of 5.0, confirming the absence of significant multicollinearity. A two-tailed *P* < 0.05 was considered statistically significant.

## 3 . Results

### 3.1 Model fit and selection of latent profiles

Latent Profile Analysis (LPA) was conducted using Mplus 8.3 software to explore social alienation among older adults with cognitive frailty, utilizing the scores of the 15 items of the GAS as explicit indicators. Models were estimated starting with one class and gradually increasing to five classes; the fit indices for each model are presented in Table 1. The results indicated a continuous downward trend in AIC, BIC, and aBIC values as the number of classes increased, suggesting that model fit improved with the addition of classes. When increasing from 3 to 4 classes, both the LMR-LRT and BLRT were statistically significant (*P* < 0.001), indicating that the 4-class model was significantly superior to the 3-class model. For instance, although a 5-class model was explored, its LMR-LRT was non-significant (*P* = 0.356), indicating no statistical advantage over the 4-class solution. Regarding classification accuracy, the Entropy value for the 4-class model was 0.850, exceeding the recommended benchmark of 0.80, which indicates clear classification boundaries. The quality and stability of the 4-class solution were further confirmed by high average latent class posterior probabilities (ranging from 0.85 to 0.91 across classes), demonstrating excellent discrimination between the profiles. Furthermore, the smallest class in the 4-class solution comprised 15.5% of the sample, satisfying the requirements for minimum class size in large-sample analyses. Ultimately, the 4-class profile was identified as the optimal model because it successfully balanced statistical fit and model parsimony, avoiding the over-stratification of a 5-class model while meaningfully capturing the clinical heterogeneity of social alienation to inform targeted psychosocial interventions.

**Table 1:** Fit indices of latent profile analysis models for social alienation among older adults with cognitive frailty (*N* = 968).

Model	AIC	BIC	aBIC	*P*		Entropy	Class proportions (%)
				LMRT	BLRT		
1-class	39,210.707	39,356.963	39,261.684				
2-class	33,922.409	34,146.670	34,000.574	<0.001	<0.001	0.945	0.390/0.610
3-class	32,744.129	33,046.393	32,849.482	<0.001	<0.001	0.908	0.206/0.358/0.436
4-class	32,491.223	32,871.491	32,623.764	<0.001	<0.001	0.850	0.155/0.235/0.265/0.345
5-class	32,311.619	32,769.891	32,471.348	0.356	<0.001	0.863	0.159/0.085/0.349/0.271/0.136

### 3.2 Characteristics of latent profiles of social alienation among older adults with cognitive frailty

**Figure 1:**
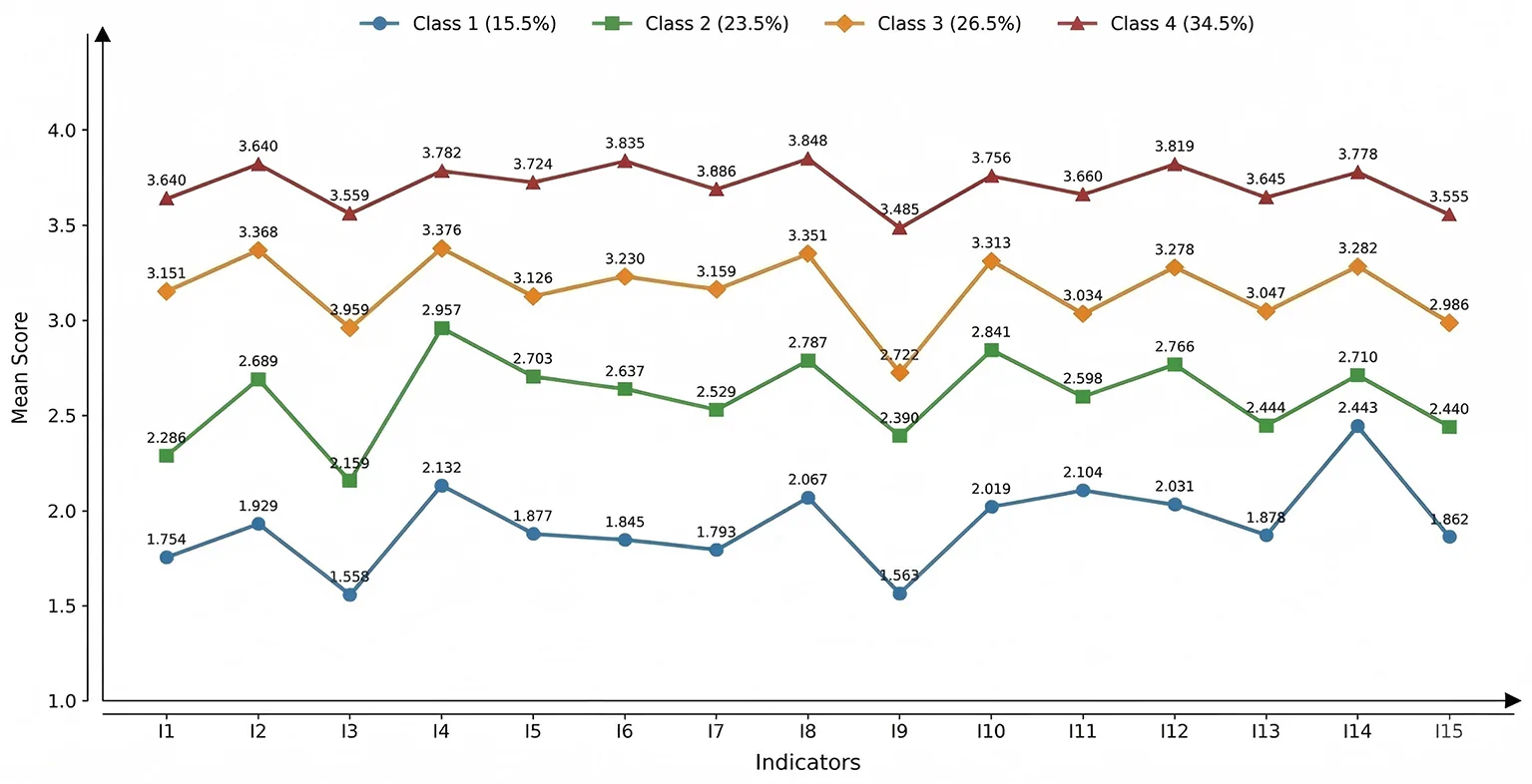
Fit statistics for latent profile models (*N* = 968).

As illustrated in Figure 1, the mean scores of the four latent profiles on the 15 items of the GAS exhibited a distinct gradient distribution. The profiles were labeled based on their scoring levels and distribution patterns. Class 1 was labeled “Social Adaptation,” accounting for 15.5% of the total sample (*N* = 150). Older adults in this group scored the lowest across all items (mean range: 1.54~2.45; total score: 28.68 ± 3.70), indicating good social integration and the absence of significant alienation. Class 2 was labeled “Mild Alienation,” comprising 23.5% of the sample (*N* = 227). Although item scores and the total score (38.87 ± 2.76) were slightly higher than those of Class 1, they remained at a relatively low level overall, reflecting mild characteristics of social alienation. Class 3 was labeled “Moderate Alienation,” representing 26.5% of the sample (*N* = 257). Item scores in this group were at a medium-to-high level (total score: 47.38 ± 2.29), suggesting the presence of noticeable social alienation issues. Class 4 was labeled “Severe Alienation,” accounting for the largest proportion of the sample at 34.5% (*N* = 334). This group exhibited the highest scores across all items (mean range: 3.50~3.86; total score: 55.70 ± 2.53), indicating that these older adults face a severe crisis of social alienation.

### 3.3 Comparison of sociodemographic and clinical characteristics among different latent profiles

The results of the univariate analysis (Table 2) indicated statistically significant differences among the four latent profiles in terms of marital status, living arrangements, social support (PSSS), depressive symptoms (GDS), and activities of daily living (BI) (*P* < 0.001). Specifically, the proportion of widowed (25.7%) and solitary-living (27.2%) older adults in the “Severe Alienation” group was significantly higher than that in the “Social Adaptation” group. Regarding physical and mental health, the “Severe Alienation” group exhibited the most severe depressive symptoms (8.21 ± 3.70), the lowest activities of daily living (86.33 ± 10.88), and the lowest level of perceived social support (52.54 ± 12.52). Furthermore, no statistically significant differences were found among the groups regarding age, gender, education level, monthly income, medical payment method, history of falls, or comorbidity of chronic diseases (*P* > 0.05).

**Table 2:** Comparison of sociodemographic and clinical characteristics among older adults with cognitive frailty across different latent profiles (*N* = 968).

Variables	Category	Social adaptation (*n* = 150)	Mild alienation (*n* = 227)	Moderate alienation (*n* = 257)	Severe alienation (*n* = 334)	χ2/F	*P*
Age (years), (*n*, %)						5.842^a^	0.441
	60–69	57 (38.0%)	76 (33.5%)	88 (34.2%)	118 (35.3%)		
	70–79	74 (49.3%)	120 (52.9%)	119 (46.3%)	168 (50.3%)		
	≥80	19 (12.7%)	31 (13.7%)	50 (19.5%)	48 (14.4%)		
Gender, (*n*, %)						0.384^a^	0.944
	Male	71(47.3%)	107 (47.1%)	118 (45.9%)	150 (44.9%)		
	Female	79 (52.7%)	120 (52.9%)	139 (54.1%)	184 (55.1%)		
Education level, (*n*, %)						5.332^a^	0.804
	Primary school or below	38 (25.3%)	65 (28.6%)	79 (30.7%)	107 (32.0%)		
	Junior high school	62 (41.3%)	88 (38.8%)	101 (39.3%)	133 (39.8%)		
	Senior high school	32 (21.3%)	50 (22.0%)	55 (21.4%)	70 (21.0%)		
	College or above	18 (12.0%)	24 (10.6%)	22 (8.6%)	24 (7.2%)		
Marital status, (*n*, %)						29.904^a^	<0.001
	Married	122 (81.3%)	168 (74.0%)	172 (66.9%)	202 (60.5%)		
	Unmarried	5 (3.3%)	10 (4.4%)	14 (5.4%)	15 (4.5%)		
	Divorced	8 (5.3%)	19 (8.4%)	24 (9.3%)	31 (9.3%)		
	Widowed	15 (10.0%)	30 (13.2%)	47 (18.3%)	86 (25.7%)		
Living arrangements, (*n*, %)	With spouse	98 (65.3%)	128 (56.4%)	115 (44.7%)	108 (32.3%)	67.273^a^	<0.001
	With children	35 (23.3%)	64 (28.2%)	85 (33.1%)	118 (35.3%)		
	Living alone	11 (7.3%)	28 (12.3%)	45 (17.5%)	91 (27.2%)		
	Nursing institution	6 (4.0%)	7 (3.1%)	12 (4.7%)	17 (5.1%)		
Monthly income (CNY), (*n*, %)						4.242^a^	0.895
	<3,000	43 (28.7%)	74 (32.6%)	92 (35.8%)	116 (34.7%)		
	3,000–5,000	67 (44.7%)	96 (42.3%)	108 (42.0%)	144 (43.1%)		
	5,000–10,000	28(18.7%)	42 (18.5%)	42(16.3%)	58(17.4%)		
	≥10,000	12 (8.0%)	15 (6.6%)	15 (5.8%)	16 (4.8%)		
Medical payment method, (*n*, %)						7.749^a^	0.257
	Employee insurance	114 (76.0%)	149 (65.6%)	168 (65.4%)	219 (65.6%)		
	Resident insurance	17 (11.3%)	38 (16.7%)	40 (15.6%)	61 (18.3%)		
	Out-of-pocket	19 (12.7%)	40 (17.6%)	49 (19.1%)	54 (16.2%)		
Comorbidity (types), (*n*, %)						9.113^a^	0.427
	None	34 (22.7%)	46 (20.3%)	63 (24.5%)	82 (24.6%)		
	1	32 (21.3%)	54 (23.8%)	49 (19.1%)	57 (17.1%)		
	2	45 (30.0%)	79 (34.8%)	75 (29.2%)	100 (29.9%)		
	≥3	39 (26.0%)	48 (21.1%)	42 (27.2%)	61 (28.4%)		
History of falls, (*n*, %)						2.763^a^	0.430
	Yes	22 (14.7%)	36 (15.9%)	46 (17.9%)	67 (20.1%)		
	No	128 (85.3%)	191 (84.1%)	211 (82.1%)	267 (79.9%)		
Scale scores (x ±s)							
Perceived social support scale		68.37 ± 8.10	61.66 ± 8.96	56.30 ± 10.91	52.54 ± 12.52	86.648^b^	<0.001
Geriatric depression scale		3.17 ± 2.04	4.67 ± 2.46	6.47 ± 3.22	8.21 ± 3.70	114.187^b^	<0.001
Barthel index		99.37 ± 1.67	96.34 ± 3.47	92.32 ± 7.03	86.33 ± 10.88	134.430^b^	<0.001

a, Chi-square test; b, One-way ANOVA.

### 3.4 Factors associated with latent profiles of social alienation

A multinomial logistic regression analysis was conducted with latent profile membership as the dependent variable, utilizing Class 1 “Social Adaptation” as the reference group. After adjusting for the forced socio-demographic covariates (age, gender, and education level), the associations for key clinical and contextual factors remained highly robust (Table 3). The adjusted model indicated that depression was a consistent risk factor across all alienation profiles, with the Odds Ratio (OR) demonstrating a continuous upward gradient as the severity of alienation increased (OR range: 1.335–1.923). Conversely, activities of daily living (OR range: 0.557–0.682) and perceived social support (OR range: 0.861–0.922) served as common protective factors. Regarding living arrangements, compared to living with a spouse, living with adult children was identified as a powerful and independent risk factor specifically associated with membership in the “Severe Alienation” profile (OR = 3.736, 95% CI: 1.672–8.348, *P* = 0.001).

**Table 3:** Multinomial logistic regression analysis of factors associated with latent profile membership of social alienation among older adults with cognitive frailty.

Variables	Mild alienation vs. social adaptation			Moderate alienation vs. social adaptation			Severe alienation vs. social adaptation		
	β **(SE)**	**OR (95% CI)**	***P***	β **(SE)**	**OR (95% CI)**	***P***	β **(SE)**	**OR (95% CI)**	***P***
Age (ref: 60–69)									
70–79	0.123 (0.254)	1.131 (0.687, 1.861)	0.628	0.285 (0.269)	1.330 (0.785, 2.253)	0.289	0.211 (0.248)	1.235 (0.759, 2.009)	0.395
≥80	0.089 (0.354)	1.093 (0.546, 2.188)	0.802	0.518 (0.377)	1.679 (0.802, 3.516)	0.169	0.101 (0.346)	1.106 (0.561, 2.179)	0.770
Gender (ref: male)									
Female	0.045 (0.231)	1.046 (0.665, 1.645)	0.846	0.111 (0.241)	1.117 (0.697, 1.792)	0.645	0.186 (0.221)	1.204 (0.781, 1.857)	0.400
Education (ref: primary or below)									
Junior high	−0.128 (0.274)	0.880 (0.514, 1.506)	0.640	−0.179 (0.288)	0.836 (0.475, 1.470)	0.534	−0.196 (0.267)	0.822 (0.487, 1.387)	0.463
Senior high	−0.302 (0.326)	0.739 (0.390, 1.401)	0.354	−0.483 (0.347)	0.617 (0.313, 1.217)	0.164	−0.432 (0.317)	0.649 (0.349, 1.208)	0.173
College or above	−0.410 (0.380)	0.664 (0.315, 1.398)	0.281	−0.700 (0.409)	0.497 (0.223, 1.107)	0.087	−0.723 (0.377)	0.485 (0.231, 1.018)	0.055
Marital status (ref: married)									
Unmarried	0.040 (0.696)	1.041 (0.266, 4.074)	0.954	−0.018 (0.754)	0.982 (0.224, 4.306)	0.981	−0.361 (0.794)	0.697 (0.147, 3.306)	0.649
Divorced	0.275 (0.646)	1.317 (0.371, 4.672)	0.670	0.018 (0.714)	1.018 (0.251, 4.128)	0.980	−0.537 (0.778)	0.584 (0.127, 2.686)	0.490
Widowed	−0.416 (1.242)	0.660 (0.058, 7.526)	0.738	−0.046 (1.290)	0.955 (0.077, 11.805)	0.972	−0.838 (1.339)	0.433 (0.032, 5.904)	0.531
Living arrangements (ref: with spouse)									
With children	0.240 (0.351)	1.271 (0.638, 2.531)	0.494	0.685 (0.386)	1.984 (0.931, 4.226)	0.076	1.318 (0.410)	3.736 (1.672, 8.348)	0.001
Living alone	−0.023 (1.174)	0.977 (0.098, 9.752)	0.984	0.951 (1.223)	2.588 (0.235, 28.461)	0.437	2.412 (1.274)	11.156 (0.918, 135.614)	0.058
Nursing inst.	−0.439 (1.394)	0.645 (0.042, 9.892)	0.753	0.840 (1.461)	2.317 (0.132, 40.640)	0.565	2.263 (1.523)	9.612 (0.485, 190.420)	0.137
Scale scores									
PSSS	−0.081 (0.014)	0.922 (0.897, 0.948)	<0.001	−0.124 (0.016)	0.883 (0.856, 0.912)	<0.001	−0.150 (0.017)	0.861 (0.832, 0.890)	<0.001
GDS	0.289 (0.056)	1.335 (1.196, 1.489)	<0.001	0.497 (0.060)	1.644 (1.462, 1.849)	<0.001	0.654 (0.063)	1.923 (1.700, 2.176)	<0.001
Barthel index (per 10-point increase)	−0.383 (0.057)	0.682 (0.610, 0.762)	<0.001	−0.510 (0.058)	0.600 (0.536, 0.673)	<0.001	−0.586 (0.059)	0.557 (0.496, 0.625)	<0.001

## 4 Discussion

This study identified four distinct latent profiles of social alienation among older adults with cognitive frailty using Latent Profile Analysis: “Social Adaptation” (15.5%), “Mild Alienation” (23.5%), “Moderate Alienation” (26.5%), and “Severe Alienation” (34.5%). The results confirm the heterogeneity of psychosocial adaptation within this population, with the predominance of the “Severe Alienation” profile signaling a severe crisis of social alienation. Depression, activities of daily living, social support, and living arrangements were identified as independently associated with latent profile membership. These findings provide empirical support for the development of targeted, stratified nursing intervention strategies.

### 4.1 Heterogeneity characteristics of social alienation among older adults with cognitive frailty

This study identified four distinct profiles of social alienation via Latent Profile Analysis, revealing significant heterogeneity in the psychosocial adaptation of older adults with cognitive frailty. This finding is consistent with prior research (29, 30), confirming that the cognitive frailty population is not a homogeneous entity. In contrast to community-dwelling older adults, who predominantly fall into low-risk groups, the “Severe Alienation” profile accounted for the largest proportion (34.5%) in this study. This suggests that severe social alienation is prevalent among older adults with cognitive frailty, warranting urgent attention. Nursing managers should prioritize including this subgroup in key visitation and dynamic monitoring lists, establishing early warning mechanisms rather than adopting indiscriminate population-based interventions.

The predominance of the “Severe Alienation” profile may be attributed to the dual decline in physical and cognitive functions associated with cognitive frailty. Research indicates that physical frailty restricts the range of activity, leading to reduced social engagement; meanwhile, declines in memory and language abilities may induce discomfort in interpersonal interactions, causing older adults to actively withdraw (31). Another study further demonstrates that this combined physical and cognitive impact creates an interaction between deteriorating health status and reduced social engagement (32). This suggests that nursing professionals should focus on this high-prevalence subgroup, as routine health education may be insufficient. Therefore, our findings suggest that future intervention protocols might benefit from combining functional rehabilitation training functional rehabilitation training (e.g., balance and gait training) with social skills training (e.g., communication encouragement and role-playing). Furthermore, creating safe, low-stress social environments (such as small-group activities) is essential to disrupt the vicious cycle of “functional decline–social withdrawal.”

Additionally, this study found that 15.5% of older adults belonged to the “Social Adaptation” profile, exhibiting low levels of alienation. This indicates that cognitive frailty does not inevitably lead to social disconnection. Studies suggest that psychological resilience and effective family social resources can buffer the negative impacts of the disease. This suggests that while focusing on high-risk groups, insights can be drawn from the “Social Adaptation” group to assist other profiles through peer support (33). Future nursing programs could explore the feasibility of identifying and cultivating older adults from the ‘Social Adaptation' group as peer supporters. By potentially organizing experience-sharing sessions or establishing a ‘buddy' system, future initiatives could test whether positive coping strategies and accessible resource networks can be systematically transmitted to high-risk groups.

### 4.2 The association of physical and mental health status with latent profiles

This study found that physical and mental health status is significantly associated with the degree of social alienation among older adults with cognitive frailty. In terms of psychological health, depressive symptoms were identified as the primary factor associated with older adults belonging to the “Severe Alienation” group (34). A potential explanation is that for older adults with already impaired cognitive function, depressive moods exacerbate cognitive load, making social interactions feel more arduous and exhausting. Simultaneously, the feelings of worthlessness and hopelessness common in depression significantly diminish the motivation to actively seek social connections, creating a vicious cycle of “cognitive frailty–depression–social avoidance.” Furthermore, depression-related somatic symptoms, such as reduced energy and sleep disorders, physiologically limit the ability to participate in social activities. This finding is consistent with Yang et al. (35), who concluded that depressive moods directly reduce social interaction activities in older adults. Moreover, research indicates that this psychological withdrawal often precedes the actual reduction in social behavior, suggesting a reciprocal reinforcing relationship between depression and social alienation (32).

Regarding physical health, Activities of Daily Living (ADL) emerged as a crucial protective factor against social alienation. Preserving good self-care abilities significantly lowers the risk of older adults falling into the “Severe Alienation” group. Research highlights that the decline in physical function is a direct precipitant of the inability to engage in social activities (36). For patients with cognitive frailty, low ADL scores imply significant difficulties in independently venturing out to participate in community activities. Additionally, the dependency on family caregivers often generates a psychological burden, making them less willing to initiate social interactions (37).

Therefore, the timely identification and intervention of depressive symptoms, alongside efforts to maintain or improve basic self-care abilities through rehabilitation training and assistive support, represent critical entry points for preventing older adults with cognitive frailty from sliding into severe social alienation.

### 4.3 The role of social context: support and living arrangements

The results of this study indicate that social contextual factors, such as social support and living arrangements, play a pivotal role in distinguishing between latent profiles of social alienation among older adults with cognitive frailty.

Social support was confirmed as a significant protective factor across all alienation profiles. This implies that higher levels of perceived social support correspond to a lower likelihood of membership in the “Severe Alienation” group. A plausible explanation is that social support networks provide emotional companionship, instrumental assistance, and informational resources, thereby directly buffering the risk of social withdrawal resulting from cognitive and physical functional decline. Research demonstrates that robust social support networks offer essential emotional and material resources, assisting older adults in coping with the stress associated with cognitive and physical deterioration (38, 39). For older adults with cognitive frailty, care from family and friends can alleviate loneliness, and enhance their sense of belonging, ultimately reducing levels of alienation (40).

Regarding living arrangements, this study revealed a noteworthy phenomenon: compared to “living with a spouse,” “living with adult children” emerged as an independent risk factor for membership in the “Severe Alienation” profile. This may be attributed to intergenerational discrepancies in lifestyle habits and values, which can lead to lower communication efficiency. Furthermore, adult children, often occupied with work, may provide “task-oriented” rather than “emotion-oriented” companionship. This finding aligns with Zhou et al. (41) who noted that care provided by spouses often encompasses deeper emotional attachment and shared lifestyle habits that are difficult for adult children to fully replicate. Furthermore, several highly plausible alternative mechanisms warrant consideration to fully contextualize this phenomenon. First, there may be an issue of reverse causation: it is highly possible that older adults are moved into their adult children's homes precisely because their cognitive and physical frailty—and accompanying severe alienation—have reached a point where independent living is no longer viable. Second, the caregiver burden placed on adult children, who often juggle demanding careers and eldercare, can create a stressful or emotionally exhausted household environment that inadvertently exacerbates the older adult's feelings of isolation. Finally, relocating to an adult child's home often signifies a profound loss of autonomy for the older adult. Forfeiting their familiar community networks, traditional family authority, and independent lifestyle can serve as a primary catalyst for severe psychological alienation, culminating in a state of “co-residence with emotional alienation” within the household.

This phenomenon may also be linked to the “Self-perceived burden” (SPB) often experienced by older adults with cognitive frailty when receiving care from their children. Due to declining functional abilities, older adults may perceive themselves as a burden to their children; consequently, they may proactively reduce interaction to avoid causing trouble, culminating in a state of “co-residence with emotional alienation” within the household. Alternatively, within multi-generational household structures, the diminution of the older adult's traditional family role and authority may adversely affect their social initiative and integration into family dynamics. This suggests that when developing interventions, nursing professionals should extend their focus beyond solitary older adults to include those living with adult children who lack spousal companionship.

### 4.4 Limitations

Several limitations of this study should be acknowledged. First, the cross-sectional design employed precludes the determination of causal relationships between variables. In particular, bidirectional associations may exist between physical and mental health status (e.g., depression, ADL) and social alienation; thus, future longitudinal studies are warranted to further verify these temporal relationships. Second, the sample was recruited via convenience sampling from only four tertiary general hospitals in Jiangsu Province. Recruiting exclusively from tertiary centers introduces a potential sampling bias, inherently skewing the cohort toward patients with more severe comorbidities, higher healthcare utilization, and heavier psychological burdens. This clinical setting bias might partially explain the unexpectedly high proportion (34.5%) of participants classified into the “Severe Alienation” profile in our study. Future studies should incorporate community-dwelling or primary care samples to provide a more balanced epidemiological perspective and validate these latent profiles across diverse care settings. Third, there is a potential conceptual overlap between our outcome measure (social alienation) and certain independent predictors, particularly depressive symptoms and perceived social support. Although these constructs are theoretically distinct—with depression representing a mood disorder and social support reflecting perceived external resources, whereas alienation captures a subjective cognitive sense of social disconnection—they inherently share overlapping psychological dimensions (e.g., feelings of withdrawal and meaninglessness). Therefore, the reported associations may partially reflect measurement overlap rather than entirely distinct explanatory pathways. Future research should employ multi-method assessments or longitudinal designs to better disentangle these intertwined psychosocial constructs. Finally, although data collection was facilitated by uniformly trained nurses, the reliance on self-report instruments introduces the potential for recall bias or social desirability bias, which may, to some extent, compromise the precision of the data.

## 5 Conclusions

This study identified four latent profiles of social alienation among older adults with cognitive frailty using Latent Profile Analysis, confirming the existence of heterogeneity in psychosocial adaptation within this population. The predominance of the “Severe Alienation” profile indicates that severe social alienation is relatively prevalent in this population. Depressive symptoms, activities of daily living, and living arrangements were identified as independent correlates distinguishing the latent profiles. These findings highlight the potential value for nursing professionals to recognize the heterogeneity of social alienation among older adults with cognitive frailty and to consider incorporating stratified assessments based on latent profiles into routine care. Furthermore, our findings provide a theoretical framework for future research to design and evaluate tailored interventions targeting the physical and mental health status, as well as the family support systems, of high-risk groups.

## Data Availability

The raw data supporting the conclusions of this article will be made available by the authors, without undue reservation.
